# The effect of neuter status on longevity in the Rottweiler dog

**DOI:** 10.1038/s41598-023-45128-w

**Published:** 2023-10-19

**Authors:** Carolynne J. Joonè, Dmitry A. Konovalov

**Affiliations:** 1 College of Public Health, Medical and Veterinary Sciences, James Cook University, Townsville, Queensland 4811 Australia; 2 College of Science and Engineering, James Cook University, Townsville, Queensland 4811 Australia

**Keywords:** Animal physiology, Endocrine system and metabolic diseases

## Abstract

Surgical sterilization or neutering of dogs is a commonly performed procedure in veterinary practices in many countries. In recent decades, concerns have been raised regarding possible side effects of neutering, including increased risk of certain neoplastic, musculoskeletal and endocrinological conditions. Considering that age serves as a significant confounding factor for some of these conditions, evaluating longevity statistics could provide valuable insights into the impact of neutering. The aim of this study was to compare longevity between neutered and sexually intact male and female Rottweilers, using electronic patient records collected by the VetCompass Australia database. Male and female Rottweilers neutered before 1 year of age (*n* = 207) demonstrated an expected lifespan 1.5 years and 1 year shorter, respectively, than their intact counterparts (*n* = 3085; *p* < 0.05). Broadening this analysis to include animals neutered before the age of 4.5 years (*n* = 357) produced similar results.

## Introduction

Surgical sterilization or neutering is well established as a means of preventing unplanned litters in dogs and cats. Pet overpopulation places a significant burden on government and non-government agencies seeking to manage these animals within acceptable animal welfare frameworks. Left unchecked, free-roaming pets pose risks to public health and safety and the state of natural ecosystems^1^. On an individual level, sterilization prevents sexual behaviors as well as roaming in the male dog and physical signs of oestrus (heat) in the female. Moreover, ovariectomy eliminates the risk of pyometra, a potentially life-threatening disorder that affects more than one in five intact female dogs by the age of ten years^2^. In addition, female dogs neutered before their first oestrus show a reduced risk of developing malignant mammary tumors; neutering up to the age of 2.5 years confers a significant protective effect^3^. Data from canine cancer registries demonstrate that mammary tumors are common, constituting up to 76% of cancer diagnoses in female dogs^4^, and that the incidence of these tumors in a dog population declines as neuter rates increase^5^.

Although the benefits of neutering are well recognized, it remains prudent to be mindful of the potential adverse effects of any elective procedure. In some European countries, elective surgeries, including neutering, are banned for pet species^6^. Veterinarians in general practice surveyed in the United Kingdom considered the main disadvantages of neutering to be weight gain, followed by either coat changes or urinary incontinence in male and female dogs, respectively^7^. In a recent study, neutered bitches were found to have twice the risk of urinary incontinence in comparison with their intact counterparts, with additional risk factors being certain breeds, increasing body weight and age, and insurance cover^8^.

Additional studies suggest that neutering may increase the risk of certain neoplastic, musculoskeletal and endocrinological conditions^9,10^, although firm conclusions might be considered elusive^11^. For some conditions, particularly neoplasia, age-at-death (lifespan) is an important confounder. As such, lifespan may be a better indicator of the overall consequences of the procedure rather than individual disease incidences^6^. To date, most studies have reported similar or increased longevity in neutered animals compared to their intact counterparts^12–19^. In contrast, Waters et al.^20^ found that the retention of ovaries for at least four years of life predisposed to extended lifespans in female Rottweilers.

The aim of the current study was to evaluate longevity and its relationship to lifetime gonadal exposure in Australian Rottweilers, using electronic patient records from the VetCompass Australia database^21^.

**Figure 1:**
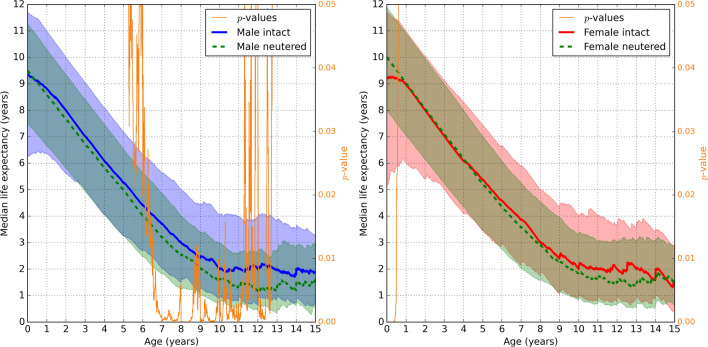
The median remaining lifespan based on age (x-axis) of (left sub-figure) male and (right sub-figure) female Rottweilers, showing sexually intact dogs (solid bold lines) compared to neutered dogs (dashed bold lines), see the legend boxes. For each solid or dashed bold line, the corresponding shaded area of the same color represents the values between the first and third quartiles. The *p*-values for Mann-Whitney U test are the thin orange lines with the right-hand-side *y*-axis.

**Figure 2:**
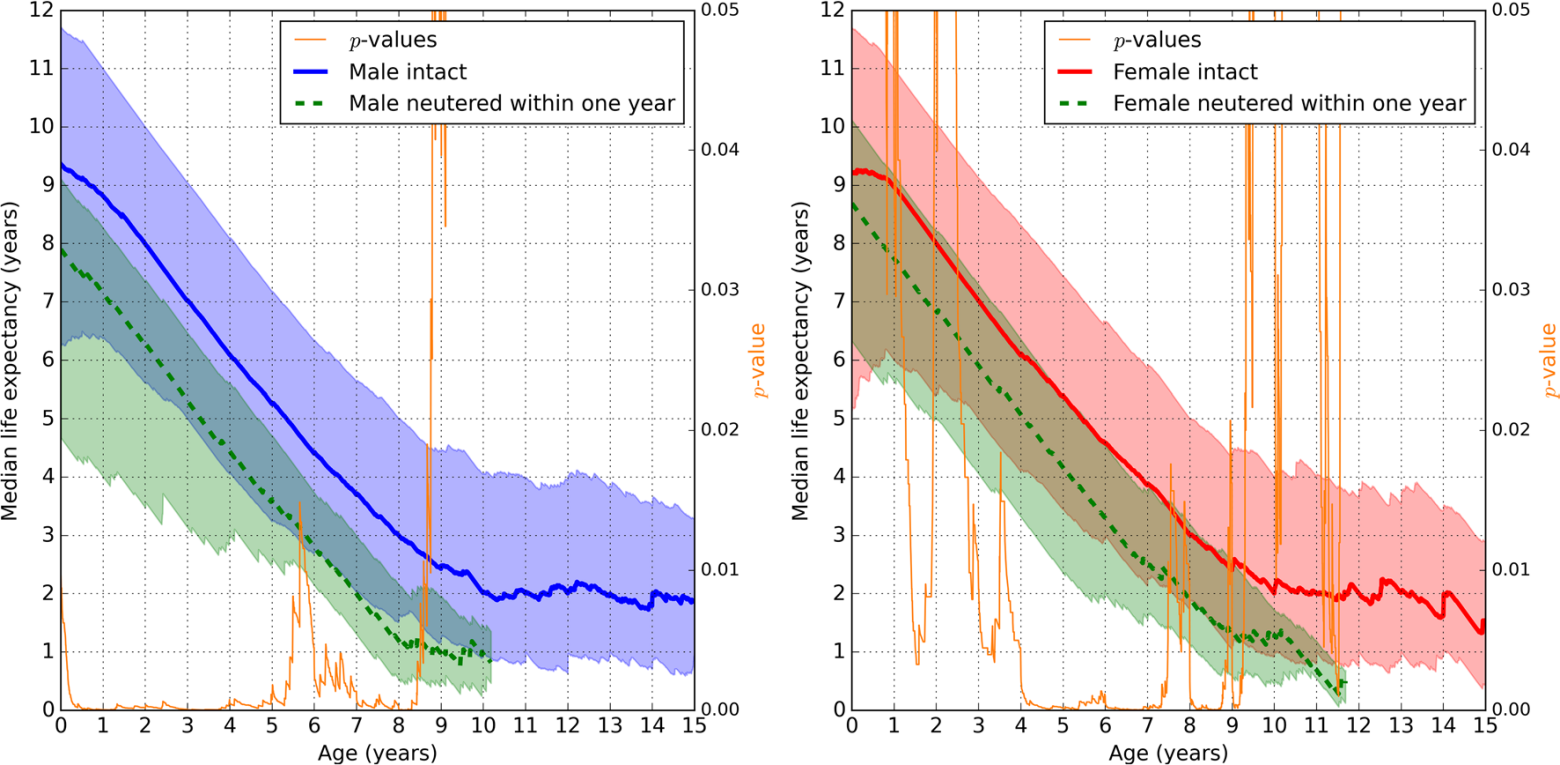
The median remaining lifespan based on age (x-axis) of (left sub-figure) male and (right sub-figure) female Rottweilers, showing sexually intact dogs (solid bold lines) compared to dogs **neutered within one year since birth** (dashed bold lines), see the legend boxes. For each solid or dashed bold line, the corresponding shaded area of the same color represents the values between the first and third quartiles. The *p*-values for Mann-Whitney U test are the thin orange lines with the right-hand-side *y*-axis.

**Figure 3:**
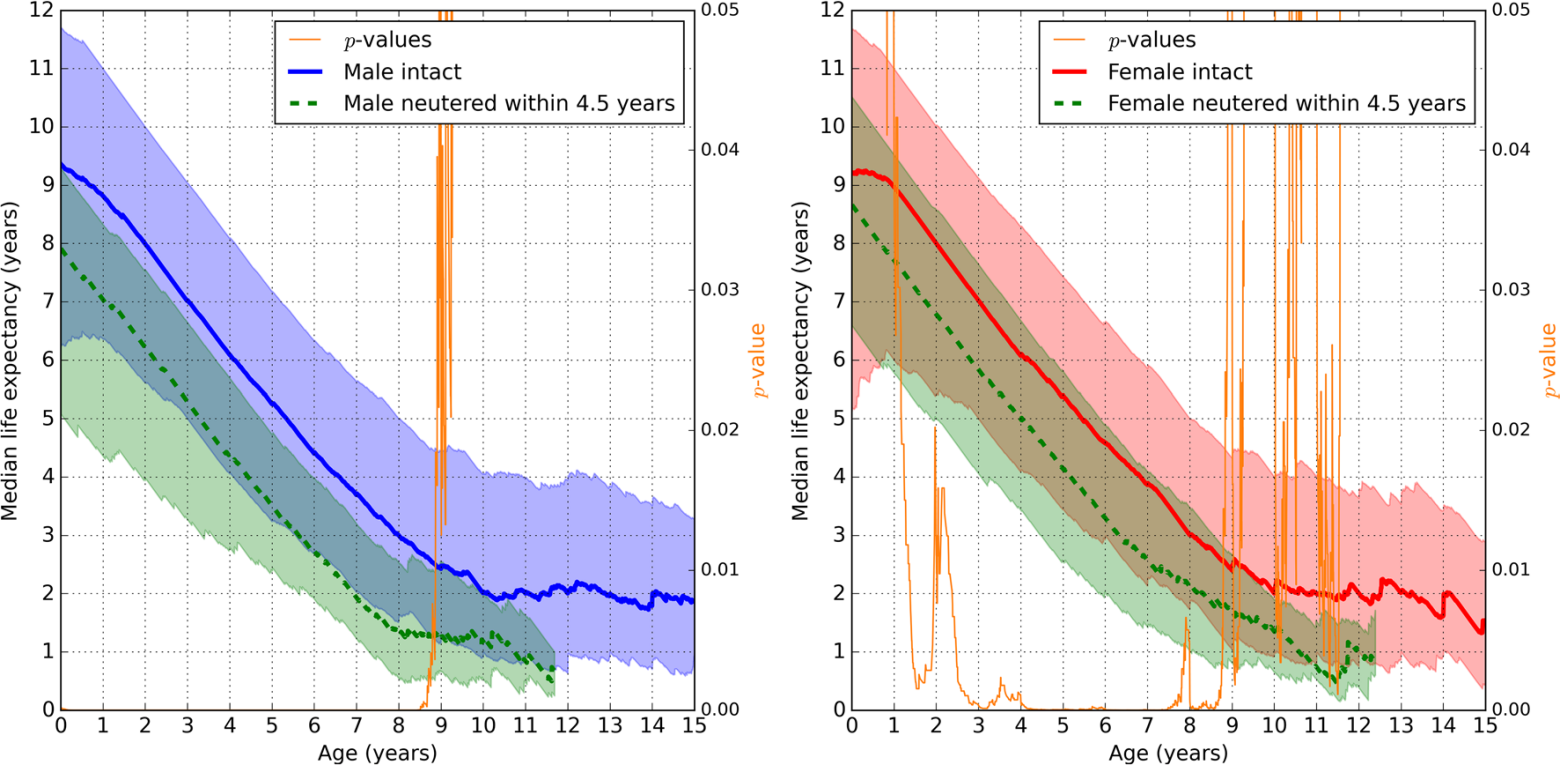
The median remaining lifespan based on age (x-axis) of (left sub-figure) male and (right sub-figure) female Rottweilers, showing sexually intact dogs (solid bold lines) compared to dogs **neutered within 4.5 years since birth** (dashed bold lines), see the legend boxes. For each solid or dashed bold line, the corresponding shaded area of the same color represents the values between the first and third quartiles. The *p*-values for Mann-Whitney U tests are the thin orange lines with the right-hand-side *y*-axis.

**Figure 4:**
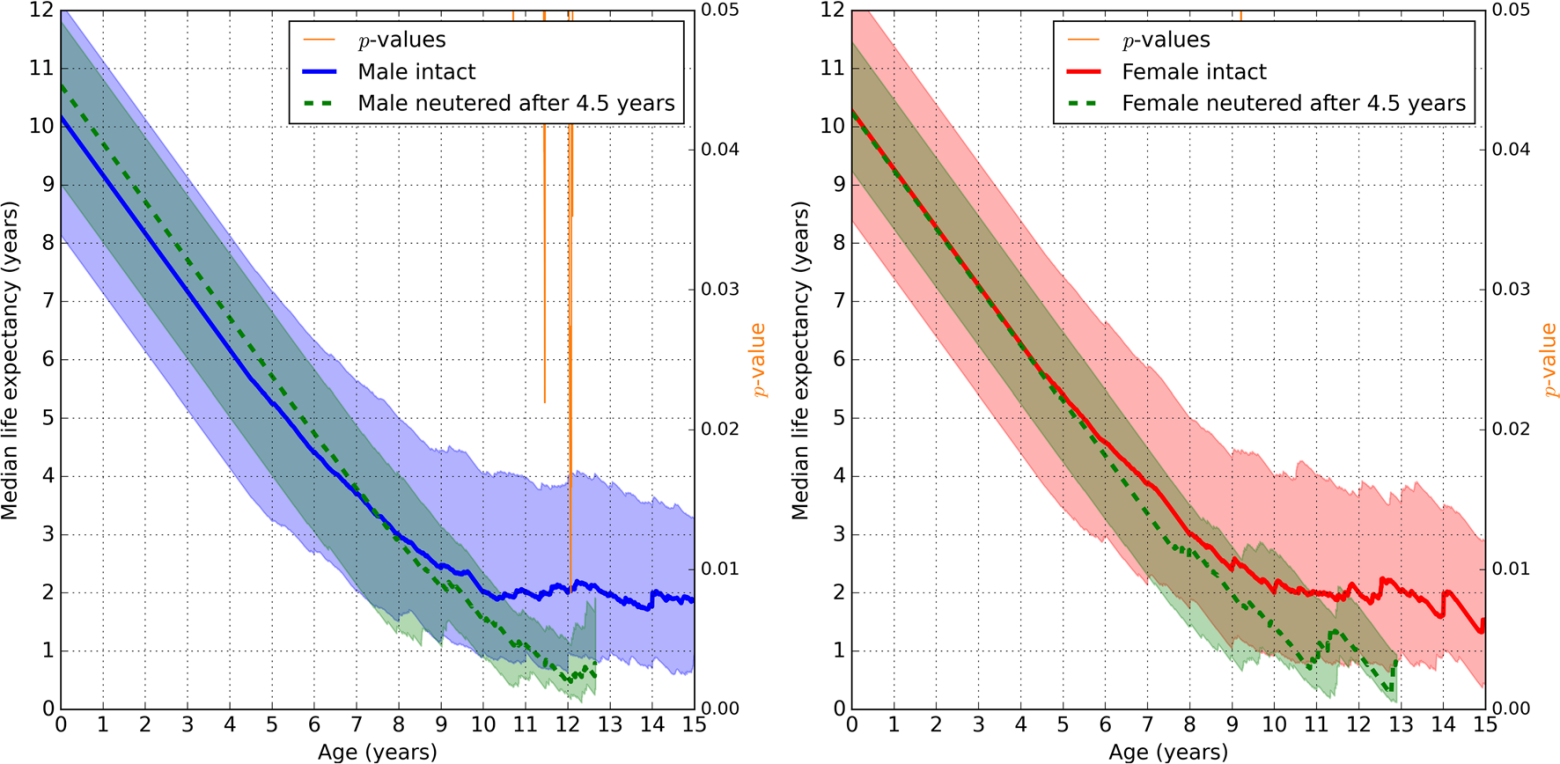
The same as Figure 3 but including only intact animals that survived to at least 4.5 years of age, and neutered animals where neutering was performed **after** 4.5 years since birth.

## Materials and methods

This study was approved by the Human Research Ethics Committee of the University of Sydney, New South Wales, Australia (protocol number: 2013/919).

Data was obtained from the VetCompass Australia database^21^. Similar to the VetCompass Programme in the UK^16^, VetCompass Australia collects and stores de-identified electronic patient records from participating veterinary practices across Australia, for research and surveillance purposes^21^. Clinical records were extracted on September 17th, 2021, based on a breed definition of “Rottweiler”. Records for each individual animal were linked based on the animal’s unique patient number or, where available, microchip number. All individuals with missing dates of birth or death were excluded from further analysis. All remaining animals were grouped according to neuter status; for the VetCompass Australia database, the digit “1” defines an animal as neutered while “0” is the default, denoting an intact animal or unrecorded neuter status. Neutered animals were further grouped according to whether the date of neuter was available, where the date of neuter was inferred from free text clinical notes and/or the charging of a neuter fee. Cut-off points for maximum age-at-neuter were preset at one and 4.5 years of age. The cut-off of one year was selected as, historically, most dogs were desexed before one year of age^22^. The cut-off of 4.5 years was selected in line with previous research that suggested a difference in lifespan between Rottweilers neutered before and after this age^20^. Data was provided in the CSV format and all required processing was performed using Python programming language.

This study examined the longevity of Rottweilers categorized by both sex and neuter status. The neutered and intact sub-populations were compared for each sex category. Similar to Tang et al.^16^, a survival analysis approach was utilized where each of the sub-populations was further sampled by whether dogs were still alive at specified ages. For these surviving sub-populations, we subsequently calculated the quartiles of the remaining lifespan values. For example, in Fig. 1, each point along the *x*-axis, therefore, portrays the remaining lifespan (median, first and third quartiles) for all dogs alive at that age, grouped according to their sex and neuter status.

The statistical significance of differences between the distributions of remaining lifespan values of intact and neutered subpopulations was determined by examining the *p*-value curves obtained from the Mann-Whitney U test^23^, where the test was performed separately at all available age points. The Mann-Whitney U test is a non-parametric test, which tests if two independent samples come from the same distribution with, consequently, the same median and quartile values. This test does not require the assumption of normal distribution. When the *p*-value curves fell below the predetermined significance level of $p < 0.05$, the null hypothesis of identical distributions was rejected for the corresponding age ranges of the *x*-axis. All *p*-values were Bonferroni corrected for multiple comparisons.

## Results

Relevant clinical records available from VetCompass Australia spanned the period July 1994 to June 2021. A total of 7,185 unique patients correlated to a breed definition of “Rottweiler”, of which 4,100 were identified as neutered. Within the subset of neutered dogs, the date of neuter was available for a total of 566 dogs. Of these, 207 dogs were neutered before the age of 1 year and 357 were neutered before the age of 4.5 years.

Figure 1 compares 3,085 intact and 4,100 neutered Rottweilers with known dates of birth and death, where age at neutering was not considered. In females destined to be neutered, the remaining lifespan median at birth (“0” on the x-axis) was around 10 years, which is significantly higher than the expected lifespan for females destined to remain intact, at around 9.2 years (right subfigure of Fig. 1). However, beyond the age of one year for females and at all ages for males, the remaining lifespan was similar or lower for neutered animals compared to their intact counterparts. For males (left subfigure of Fig. 1), based on Mann-Whitney’s *p*-curves< 0.05 (Bonferroni corrected), the distribution medians and quartiles were significantly different between 5.5 and 12.5 years of age, where the medians and 3rd quartiles were typically 0.3-0.5 and 0.5-1.0 years larger for intact males, respectively. For females (right subfigure of Fig. 1), the *p*-value curves> 0.05 suggested that the distribution quartiles were not significantly different beyond one year of age, even though the values of the 3rd quartiles were up to one year larger for intact females.

Figure 2 compares male and female Rottweilers neutered before the age of 1 year, with their intact counterparts. Similarly, Fig. 3 compares male and female Rottweilers neutered before the age of 4.5 years, with their intact counterparts. Figures 2 and 3 show that the third quartile curves of neutered dogs were below (for males) or comparable (for females) to the median curves of intact dogs, implying that at least 50% of intact Rottweilers lived longer than 75% of neutered Rottweilers. The differences were statistically significant in males up to approximately 9 years of age and in females within the range of 1-9 years. Figures 2 and 3 are qualitatively comparable indicating that the presented results are not specific to a particular choice of neutering age.

Finally, Rottweilers neutered at any time after the age of 4.5 years were compared to intact animals that survived to at least 4.5 years of age. The difference in median life expectancy values was not significant, see Fig. 4. Beyond approximately 9 years of age, neutering appears to be associated with a decrease in lifespan but could not be statistically confirmed due to the small number of dogs neutered after 4.5 years of age and still alive beyond 9 years old. At (and up to) the cut-off age of 4.5 years, the median life expectancy values of neutered and intact sub-populations were similar (*p*>0.05, Bonferroni corrected) (Fig. 4), confirming that the two sub-populations were comparable up to this point.

## Discussion

The findings of the current study indicate that intact Australian Rottweilers registered on the VetCompass Australia database have a longer lifespan than their neutered counterparts. For example, when neutering was performed before the age of 4.5 years, intact male and female Rottweilers lived about 1.5 years and 1 year longer, respectively, than neutered Rottweilers, across all applicable ages. In contrast, previous studies, involving a variety of breeds, have linked neutering to an increase in lifespan^6^. Further study involving additional breeds is warranted to clarify whether the findings of the current study are unique to the Rottweiler breed.

Waters et al.^24^ suggest that longevity studies where animals are classified as neutered or intact at the time of death, regardless of age at neutering, may lead to erroneous conclusions regarding the impact of neutering. In the current study, the expected lifespan at birth for a Rottweiler dog destined to be neutered was comparable (for males) or higher (for females) than dogs destined to remain intact (Fig. 1). However, all neutered dogs must survive until, at least, the age at which they are neutered. By examining the life expectancy quartile curves as a function of age, we identified a neutering preselection bias that inflated the median lifespan of neutered females (at birth) by 0.75 years, with the expected lifespan of intact and neutered males being similar (at birth). That bias disappeared after 1-2 years of age; the life expectancy of intact and neutered females became identical, while the life expectancy of intact males exceeded their neutered counterparts from around 6 years of age (Fig. 1).

Traditionally, elective neutering is performed between the ages of around 6 months and 1 year^22^. In response to, in particular, a purported increased risk of osteosarcoma in Rottweilers neutered before one year of age^19^, some breeders and dog owners may elect to neuter non-breeding Rottweilers beyond this age. To evaluate this strategy, the age of 1 year was selected as a cut-off of interest, see Fig. 2. Interestingly, from our dataset, the expected lifespan of a 1-year-old, neutered Rottweiler would be approximately 12 to 15% less than an intact Rottweiler of the same age. The reasons behind this finding are currently speculative. Several factors will influence the lifespan of a dog, including genetic predisposition, nutrition, exercise, healthcare, and environmental factors. Screening for genetic disorders and selecting healthy breeding pairs may help reduce the prevalence of hereditary health conditions in Rottweilers and contribute to their longevity^25^. Moreover, a health-related neutering bias could exist where healthier dogs are selected for breeding and not neutered. Hence, by this selection process, some intact dogs would live longer than their neutered counterparts but not necessarily due to non-neutering as such.

Although diet and exercise play an important role in determining body weight, a link between neutering and obesity is well established^7,9,10^. In a study utilizing electronic patient records in the United Kingdom, only 7% of Rottweilers were listed as overweight or obese. However, as a clinical diagnosis of obesity may have been easy to overlook in light of more pressing concerns, this proportion was considered likely to be an underestimate^18^. Importantly, bodyweight has been shown to exert a significant negative effect on lifespan^26^. Further research is required to establish how bodyweight may have influenced the findings of the current study.

Waters et al.^20^ found that female Rottweilers were three times more likely to live to an extremely old age compared to males, a survival advantage that was lost in females neutered before the age of four years. This finding suggests that the timing of neutering, particularly in females, may play a crucial role in determining potential benefits or drawbacks associated with the procedure. Research on female Rottweilers that excluded extremely old aged individuals showed that dogs neutered before the age of 4.5 years had significantly shorter life expectancy than those neutered at or after the age of 4.5 years^20^. For this reason, 4.5 years was selected as the second cutoff of interest in the current study, see Fig. 3. Our results confirm the findings of Waters et al.^20^ for female Rottweilers, in that the expected lifespan was significantly longer in intact females than those neutered before 4.5 years of age. Although not statistically significant, the median remaining lifespan of female Rottweilers neutered after the age of 4.5 years is lower than that of intact individuals, from around 6 years of age. Similarly, while initially higher, the median remaining lifespan in males neutered after the age of 4.5 years appears to decrease at a faster rate than intact males (Fig. 3). Further research using a larger sample size is warranted to establish whether neutering, at any age, has a negative impact on life expectancy in this breed.

Interestingly, in the current study, an even greater negative effect on expected lifespan occurred in male Rottweilers compared to females, a result not reported by Waters et al.^20^ despite the inclusion of male Rottweilers in their original dataset. Our findings fail to support this group’s conclusions regarding the protective effects of ovaries on the ageing female, unless such an effect could also be attributed to testes in the ageing male. In their study, Waters et al.^20^ considered the influence of extraneous factors on the apparent association between age at neuter and longevity demonstrated by their findings. For example, the owner of a female Rottweiler known to survive to an extremely old age may purposely select for breeding, and hence delay neutering, her female offspring-which may themselves be genetically predisposed to an extended lifespan. Alternatively, dogs of substandard conformation or physical health may be removed from the breeding pool, and neutered, early in life. In their investigation, Waters et al.^20^ found no evidence that such factors played a significant role. Further investigation of these factors was not possible within the current study. Moreover, it remains possible that neutering at any age, ultimately, has a negative impact on the expected lifespan in this breed. If so, this would suggest that selection processes carried out by breeders and dog owners during the early years of their dogs’ lives have relatively little impact on longevity of this breed.

The median lifespan of intact Australian Rottweilers in the current study was a little over nine years of age for both males and females. Similarly, the median age at death of Rottweilers in the UK has been reported as 9.0 years of age^18^ and 9.8 years of age^13^, while Rottweilers in North America reportedly lived to a median age of 9.5 years^14^.

A limitation of the current study was the uncertainty of neuter statuses recorded as the default “0” (intact or not recorded), for which a proportion may have consisted of neutered animals for which the electronic patient record was incomplete. However, the removal of these individuals, if they could be identified, would be unlikely to impact the findings of the study or would serve to widen the demonstrated lifespan advantage of intact over neutered Rottweilers. A second limitation was the modest sample size for neutered dogs (compared to intact) for which the date of neuter could be traced. Given ongoing interest in the duration of gonadal exposure in dogs in relation to lifespan and disease incidence^24^, veterinary practices participating in databases such as VetCompass should be alerted to the importance of recording this detail.

## Conclusion

In conclusion, the findings of the current study suggest that neutering prior to 4.5 years of age has a negative impact on lifespan in the Rottweiler breed. Further research to establish reasons for this finding is warranted. In addition, similar studies on different breeds of dogs may clarify whether this effect is unique to this particular breed. Due to the limited sample size for dogs neutered after 4.5 years of age, the existence of an optimal age for neutering a Rottweiler dog remains to be established and may not exist for this breed. Nevertheless, veterinary clinicians are encouraged to keep in mind the well-established benefits of neutering when making recommendations to owners of Rottweiler dogs.

## Data Availability

The data that support the findings of this study are available from VetCompass Australia but restrictions apply to the availability of these data, which were used under license for the current study, and so are not publicly available. Data are however available from the corresponding author upon reasonable request and with permission of VetCompass Australia.
